# Predicting Academic Alienation From Emotion Dysregulation, Social Competence, and Peer Relationships in School-Attending Girls: A Multiple-Regression Approach

**DOI:** 10.3389/fpsyg.2021.755952

**Published:** 2021-12-24

**Authors:** Zohreh Vafa, Morteza Azizi, Mojtaba Elhami Athar

**Affiliations:** ^1^Department of Psychology, Islamic Azad University, Sarab Branch, Sarab, Iran; ^2^School of Behavioral Sciences and Mental Health (Tehran Institute of Psychiatry), Iran University of Medical Sciences, Tehran, Iran

**Keywords:** school alienation, emotion dysregulation, social competence, peer relationships, adolescents

## Abstract

School alienation (SA) refers to a collection of negative attitudes toward the social and academic realms of schooling consisting of cognitive and affective components. The current study was designed to examine whether emotion dysregulation, social competence, and peer problems predict school alienation. In this vein, 300 school-attending adolescents in Sarab were recruited and completed difficulties in emotion regulation scale (DERS), academic alienation questionnaire (AAQ), social competence test (SCT), and index of peer relations (IPR) measures, but 280 (M age = 16.35; *SD* = 0.82; 46% girls) completed data were gathered. The results of hierarchical multiple regression indicated that school alienation was significantly predicted by emotion dysregulation, social competency, and peer problems. In conclusion, our findings suggest that school psychologists and other clinicians design interventions to improve the students’ shortcomings in emotion regulations, social competency, and peer relationships domains.

## Introduction

School alienation (SA) includes an assortment of negative attitudes toward social and academic realms of schooling and consists of cognitive and affective components. Accordingly, the cognitive dimension of SA is related to the students’ evaluations of the school environment, while the affective component relates to their feelings toward schooling (Hascher and Hadjar, 2018). SA consists of three interrelated core components, including (1) learning, which is the principal goal of schooling; (2) teachers as instructors and mentors representing the school authority (professional level) and constitute a part of the social environment, and (3) classmates as the social peer community (Hascher and Hadjar, 2018). A school alienated student experiences isolation from a group or activity to which she/he belongs and should be involved (Mann, 2001). SA usually happens in the adolescence period, which is considered a risky developmental stage. For instance, the prevalence and onset of depression and anxiety problems increase significantly during this age period. These issues can detrimentally influence adolescents’ psychological functioning and lead to adjustment problems in school, peer relationships, and general social and emotional malfunctions. All these events may make the adolescents vulnerable to SA (Kaufman et al., 2001; Beesdo et al., 2009; Ilioi and Golombok, 2014). Alienation in adolescence is expressed through unfavorable behaviors, such as violence, vandalism, school aversion, and deviant behavior. In the school environment, alienated students tend to devalue school activities or consider themselves incapable of engaging in school activities (Trusty and Dooley-Dickey, 1993; Brown, 2004). Additionally, SA is associated with manifold adverse consequences for individuals, classrooms, schools, and societies, which is manifested in behavioral disengagement from learning, deviant behavior, problematic educational trajectories, school drop-out, and high risks of incomplete basic education (e.g., Archambault et al., 2009; Sutherland, 2011; Studsrød and Bru, 2012; Avci and Çelikkaleli, 2016). All these taken into account, it is necessary to examine the factors leading to alienation from school. In this regard, several predictors have been found to have a role in the development of SA, such as gender, socio-cultural background, and academic aspirations (Green-Demers et al., 2008), general decrease in academic motivation during adolescence (Jacobs et al., 2002), negative experiences in roles as students, social relationships in school (e.g., between students and their teachers; Legault et al., 2006), lack of autonomy in the learning environments (Assor et al., 2002, 2005), relationships with peers and parents (Legault et al., 2006). Therefore, it can be concluded that SA is influenced by a combination of social and psychological factors. Notwithstanding, our review indicated that the literature on the SA has mostly examined the role of social and structure/organizational culture of schools on the development of SA, while few studies have examined the role of psychological factors. For instance, Tarquin and Cook-Cottone (2008) studied a total sample of 351 undergraduate students and found a moderate negative correlation between self-concept and student alienation. Similarly, a study by Buzzai et al. (2021) supported the roles of mastery orientation and learned helplessness in the relationship between students’ perceptions of school alienation and academic achievement. In another study, Morinaj and Hascher (2019) examined the association between school alienation and student well-being over a 1-year interval. Their results indicated that alienation from learning had a significant negative influence on positive views of the school and school enjoyment. Similarly, alienation from teachers negatively predicted positive attitudes to school, while it positively predicted worries and social problems in school. In the same vein, alienation from classmates negatively affected future positive attitudes toward school and contributed to the pervasiveness of social problems in school. Notwithstanding, still, there are several overlooked critical psychological variables that can have significant role in the development of SA.

One of the psychological factors that is likely to have a role in the development of SA is emotion dysregulation (ED). ED refers to an inability to use healthy strategies to diffuse or moderate negative emotions, which interferes with goal-directed activity (e.g., Rolston and Lloyd-Richardson, 2017; Thompson, 2019). Emotions play a crucial role in academic settings and academic performance. Normal emotion regulation is central to the individual’s personal and educational well-being (Guzmán-González et al., 2016). Furthermore, emotions work as a control mechanism alerting individuals about the significance of the actual setting to steer their reaction to adapt to it (Mikolajczak and Van Bellegem, 2017). Poor emotion regulation leads to difficulty in controlling impulse and emotion and experiencing unpleasant emotions, which is a risk factor for peer acceptance and school maladjustment (Andrei et al., 2015). In this regard, Schelble et al. (2010) indicated that emotion regulation ability is significantly associated with academic resilience (i.e., normal functioning despite having experienced maltreatment). In another study, Hughes et al. (2010) investigated emotion regulation strategy use in a sample of 21 clinic-referred children and adolescents who presented with school refusal and were diagnosed with at least one anxiety disorder. Their results indicated that children and adolescents presenting with school refusal reported less adaptive emotion regulation strategy use than nonclinical children and adolescents; that is, compared to the nonclinical sample, the school refusal adolescents reported less use of cognitive reappraisal and greater use of expressive suppression to regulate their emotions. Similarly, Kitahara et al. (2020) suggested that school adjustment was highest among students who used the reappraisal emotion regulation mechanism, and it had positive direct and indirect effects on school adjustment. Kitahara et al. (2020) also indicated that reappraisal is an effective emotion regulation strategy, promoting the likelihood of receiving social support and enhances school adjustment.

In addition to ED, Social competence (SC) is another factor that is likely to have a significant role in SA. Social competence is the ability to handle social communications effectively, get along well with others, form and maintain close relationships, and respond in adaptive ways in social settings (Orpinas, 2010). SC helps one develop positive relationships with other students in classroom interactions (Elijah and Madeira, 2013). On the other hand, the lack of solidified SC would result in chaotic relationships with students and parents, which is among the multiple factors leading to SA (Legault et al., 2006). Both poor ED and SC influence interpersonal and peer relationships negatively. Gowing (2019) indicated that students considered peer relationships (PR) as the most valued aspect of their school experience, and peer relationship was found as a resource that builds connectedness to school. In addition, Schmidt et al. (2019) showed that feeling related to peers is associated with high positive emotions at school and home, which indicates the importance of peer relatedness in promoting positive well-being experienced in all life situations, especially in school.

In sum, our review indicates that ED, SD, and PR are likely to be associated with SA. Notwithstanding, to our knowledge, no study has examined the influence of these factors on SA. Therefore, this study was designed with a sample of 300 school-attending adolescents in Iran and aimed to examine whether SA is predicted by ED, SD, and PR.

## Materials and Methods

### Participants

Participants were 300 school-attending girls aged 15–18 years old who were recruited from high schools in Sarab between March 2020 to June 2021. Specifically, one district of Sarab city was selected randomly, and we then randomly chose two high schools from the selected districts. Finally, six classes (grades 10–12) from these schools were selected randomly, and the questionnaires were distributed to 300 students in the classes, but 280 (*M*age = 16.35; *SD* = 0.82) participants completed questionnaires (response rate: 93.33%).

### Procedure

Prior to data gathering, the participants and their teachers were explained about the aims and process of the study. They were then informed about the data’s confidentiality and that it would only be used for the present study. After providing their informed consent, participants were asked to fill out the measures, while they could ask questions from the data gatherer if they needed. The participants were also asked to write down their e-mail addresses should they want to receive the result of their completed questionnaire. The participants completed the questionnaires in their classroom during a one-hour session under the supervision of a specially trained research assistant (master-level student). This study was approved by the ethics committee of the Islamic Azad University, Sarab Branch. Also, approval was provided by the Iran Ministry of Education and boards of each school.

### Measures

#### Difficulties in Emotion Regulation Scale

The difficulties in emotion regulation scale (DERS; Gratz and Roemer, 2004) is a 36-item self-report questionnaire that assesses EDs. The DERS items load on six subscales, including Lack of Emotional Awareness (6 items), Lack of Emotional Clarity (5 items), Difficulties Controlling Impulsive Behaviors When Distressed (6 items), Difficulties Engaging in Goal-Directed Behavior When Distressed (5 items), Nonacceptance of Negative Emotional Responses (6 items), and Limited Access to Effective ER Strategies (8 items). Participants rate items on a 5-point scale ranging from 1 (*almost never*) to 5 (*almost always*). Subscale scores are obtained by summing the corresponding items. Besharat and Bazzazian (2013) supported the internal consistency and validity of the Persian version of DERS. Cronbach’s alpha for the DERS scores in the current study can be retrieved from Table 1.

**Table 1:** Descriptive statistics for study variables (*n* = 280).

Measures	Mean (SD)	Skewness	Kurtosis	*α*
School Alienation	33.91(12.25)	1.29	1.01	0.83
Feeling anomalous	12.17(4.24)	0.82	0.15	0.75
Feeling disability	12.72(6.14)	1.43	0.72	0.82
Feeling isolated	9.01(3.40)	1.35	1.58	0.79
DERS Total Score	79.97(21.92)	1.40	1.58	0.85
Nonacceptance	13.07(5.95)	1.16	0.02	0.80
Lack awareness	13.48(6.15)	1.03	−0.32	0.71
Goal	11.61(4.26)	0.73	0.54	0.77
Impulse	13.83(5.83)	0.49	−0.80	0.67
Strategies	16.89(7.40)	1.28	0.76	0.85
Clarity	11.06(4.46)	1.26	0.80	0.65
Social Competence	223.50(47.58)	−0.42	−0.99	0.86
Behavioral skills	166.42(45.87)	−0.40	−1.15	0.83
Cognitive skills	26.18(8.87)	−0.26	−0.32	0.88.
Emotional skills	16.08(2.70)	−0.82	0.76	0.79.
Motivational skills	14.80(4.03)	−1.01	0.30	0.85
IPP	51.52(35.36)	1.21	0.80	0.91

*SD, Standard Deviation; α, Cronbach’s Alpha Coefficient; DERS, Difficulties in Emotion Regulation Scale; Goal, Difficulties Engaging in Goal-Directed Behavior; Impulse, Difficulties Controlling Impulsive Behaviors; Nonacceptance, Nonacceptance of Negative Emotional Responses; Strategies, Limited Access to Emotion Regulation strategies; Clarity, lack of emotional clarity; IPP, Index of Peer Relations*.

#### Academic Alienation Questionnaire

Academic alienation questionnaire (AAQ; Dillon and Grout, 1976) includes 17 items which are rated on a 5 point Likert scale ranging from 1 (*completely false*) to 5 (*completely true*) and load on three subscales, including feeling anomalous (6 items), feeling disability (6 items), and feeling isolated (5 items). Qalavandi et al. (2014) supported the psychometrics of AAQ in Iran on 289 high school students in Qom, and Cronbach’s alpha coefficient 0.86, 0.81, 0.81, 0.77 were found for AAQ Total scale, isolation, disability, and anomalous, respectively. Cronbach’s alpha for the AAQ scores in the current study can be retrieved from Table 1.

#### Social Competence Test

Social competence test (SCT; Felner, 2002) includes 47 questions and four dimensions of behavioral skills, emotional skills, cognitive skills, and motivational skills. Items are rated based on a seven-point Likert scale from score 1 (*strongly disagree*) to 7 (*strongly agree*). Perandin (2006) supported the psychometrics of SCT to be used in Iran. Cronbach’s alpha for the SCT scores in the current study can be retrieved from Table 1.

#### Index of Peer Relations

The index of peer relations (IPR) was developed by Hudson et al. (1993) and is a 25-item self-report scale to measure the quality of peer relations from adolescence through adulthood. The IPR uses a 7-point Likert-type scale with scores ranging from 1 (“*true none of the time*”) to 7 (“*true all of the time*”). The items are worded both positively and negatively to control for response set bias. Scoring is reversed for positively worded items. The higher the total score, the greater the likelihood of peer relation problems. Kimiaee et al. (2011) examined the psychometrics of the Persian IPR, and the results yielded acceptable psychometrics for the measure. Cronbach’s alpha for the IPR score in the current study can be retrieved from Table 1.

#### Data Analyses

First, in the data screening process, to include as many cases as possible, missing values were examined using the series mean method in SPSS 20; also, we used the Boxplot method to deal with outliers, which resulted in a sample with 280 students. We implemented SPSS 20 software for data entry and statistical analyses. First, descriptive information was calculated for the variables, which are retrievable from Table 1. Then, data were analyzed using Pearson correlation coefficient and hierarchical multiple regression analysis. For hypothesis testing, we considered *p* < 0.05 as indicating statistically significant results.

## Results

First, Pearson’s correlation was calculated between all study variables. Results indicated that ED scores had a significant positive correlation with SA scores, while the SC scores were significantly and negatively associated with SA. Also, our results showed that there were no significant associations between SA and peer relationships problems (see Table 2 for more information).

**Table 2:** Correlations between DERS, SC, IPP, and SL Scores (*n* = 280).

	Feeling anomalous	Feeling disability	Feeling isolated	School Alienation Total score
DERS Total Score	0.34^**^	0.54^**^	0.50^**^	0.53^**^
Nonacceptance	0.26^**^	0.49^**^	0.34^**^	0.41^**^
Lack awareness	0.03	0.18^**^	0.11	0.13^*^
Goal	0.21^**^	0.11	0.16^**^	0.17^**^
Impulse	0.33^**^	0.43^**^	0.40^**^	0.44^**^
Strategies	0.18^**^	0.46^**^	0.42^**^	0.47^**^
Clarity	0.34^**^	0.38^**^	0.39^**^	0.42^*^
Social Competence	−0.23^**^	−0.34^**^	−0.28^**^	−0.33^**^
Behavioral skills	−0.14^*^	−0.22^**^	−0.17^**^	−0.21^**^
Cognitive skills	−0.20^**^	−0.33^**^	−0.33^**^	−0.33^**^
Emotional skills	−0.44^**^	−0.56^**^	−0.45^**^	−0.56^**^
Motivational skills	−0.35^**^	−0.48^**^	−0.35^**^	−0.46^**^
IPP	0.10	0.11	0.09	0.12

*DERS, Difficulties in Emotion Regulation Scale; Goal, Difficulties Engaging in Goal-Directed Behavior; Lack awareness, Lack of Emotional Awareness; Impulse, Difficulties Controlling Impulsive Behaviors; Nonacceptance, Nonacceptance of Negative Emotional Responses; Strategies, Limited Access to Emotion Regulation strategies; Clarity, lack of emotional clarity; IPP, Index of Peer Relations*.

** *p < 0.001; and*

* *p < 0.05*.

To examine whether Emotion Dysregulation, Social Competence, and Peer Relationships predict Academic Alienation, a three-stage hierarchical multiple regression was conducted with SA as the dependent variable (Table 3). DERS subscales were entered at stage one of the regression. The SC subscales were entered at stage two and IPP at stage three. The results of each step in the regression analysis and individual beta coefficients with associated significance are provided in Table 3. The first model, including DERS subscales, was statistically significant, *R*^2^ = 0.303, *F*(6, 273) = 19.74, *p* < 0.001. DERS subscales were significantly positively associated with SL and explained 30% of the variance in the dependent variable. Introducing the SC variables in the second step explained an additional 26% of variation in SA and this change in *R*^2^ was significant, *R*^2^ = 0.563, *F*(4, 269) = 39.95, *p* < 0.001. Similarly, in the significant third step of the model, IPP contributed independently with 0.010% to SA, *R*^2^ = 0.572, *F*(1, 268) = 6.16, *p* < 0.001. All predictor variables together accounted for 57.2% of the variance in SA scores. However, lack of awareness (*β *= −0.094, *p* < 0.05), limited access to emotion regulation strategies (*β *= 0.128, *p* < 0.05), lack of emotional awareness (*β *= 0.179, *p* < 0.01), cognitive skills (*β *= −0.193, *p* < 0.001) emotional skills (*β *= −0.309, *p* < 0.001), and IPP (*β *= 0.109, *p* < 0.05) had statistically significant beta coefficients among the variables.

**Table 3:** Hierarchical regression of SA on DERS, SC, and IPP (*n* = 280).

		Unstandardized coefficients		Standardized coefficients	
Step	Variable	*B*	*SE*	*β*	*p*	*R* ^2^	*R*^2^ *change*	*F*	*P*
1	Nonacceptance	0.337	0.137	0.164	0.001	0.303	0.303	19.72	0.001
	Lack awareness	0.095	0.111	0.048	0.014				
	Goal	0.476	0.150	0.166	0.393				
	Impulse	0.484	0.138	0.231	0.002				
	Strategies	0.311	0.113	0.188	0.001				
	Clarity	0.127	0.209	0.046	0.006				
2						0.563	0.26	39.95	0.001
	Nonacceptance	0.175	0.111	0.085	0.115				
	Lack awareness	−0.142	0.092	−0.071	0.124				
	Goal	0.152	0.122	0.053	0.214				
	Impulse	0.149	0.118	0.071	0.207				
	Strategies	0.201	0.091	0.122	0.028				
	Clarity	0.475	0.175	0.173	0.007				
	Behavioral skills	−0.009	0.011	−0.032	0.448				
	Cognitive skills	−0.247	0.062	−0.179	0.000				
	Emotional skills	−1.398	0.211	−0.309	0.000				
	Nonacceptance	−0.903	0.140	−0.297	0.000				
3						0.572	0.010	6.159	0.014
	Nonacceptance	0.187	0.110	0.091	0.091				
	Lack awareness	−0.188	0.093	−0.094	0.044				
	Goal	0.128	0.122	0.045	0.294				
	Impulse	0.109	0.118	0.052	0.357				
	Strategies	0.212	0.091	0.128	0.020				
	Clarity	0.492	0.174	0.179	0.005				
	Behavioral skills	0.001	0.012	0.003	0.946				
	Cognitive skills	−0.266	0.062	−0.193	0.000				
	Emotional skills	−1.401	0.209	−0.309	0.000				
	IPP	0.038	0.015	0.109	0.014				

*DERS, Difficulties in Emotion Regulation Scale; Goal, Difficulties Engaging in Goal-Directed Behavior; Lack awareness, Lack of Emotional Awareness; Impulse, Difficulties Controlling Impulsive Behaviors; Nonacceptance, Nonacceptance of Negative Emotional Responses; Strategies, Limited Access to Emotion Regulation strategies; Clarity, lack of emotional clarity; IPP, Index of Peer Relations*.

## Discussion

In the current study, we aimed to examine whether Emotion Dysregulation (ED), Social Competence (SC), and Peer Relationships (PR) predict School Alienation (SA). Toward this aim, hierarchical multiple regression was conducted, and the results indicated that ED, SC, and PR significantly predicted SA.

Consistent with our results, previous studies have indicated the negative influence of ED on SA (Schelble et al., 2010; Kwon et al., 2017; Kitahara et al., 2020). Accordingly, emotions have a significant role in performance in academic settings, and emotion regulation abilities greatly enhance one’s performance in academic situations (Guzmán-González et al., 2016). Normal ER is also associated with greater social functioning, such as peer acceptance and higher peer status. Therefore, students with poor ER would experience more peer problems and interpersonal difficulties, which are associated with SA (Buckley and Saarni, 2014; Kitahara et al., 2020). In addition, emotion regulation abilities are associated with higher levels of behavioral and cognitive engagement, openness to learning new materials, and greater academic performance. On the other hand, poor emotion regulation leads to lower levels of motivation to learn, rigid learning strategies, external regulation of learning, and impaired academic performance, all of which are strongly related to SA (Burić and Sorić, 2012; Ahmed et al., 2013). Additionally, Filippello et al. (2018) examined the association between cognitive emotion regulation and school refusal and found that catastrophizing and rumination positively predicted school avoidance. The authors inferred that students might catastrophize school situations significantly, so they do not feel comfortable going to school. Similarly, students who cling to the rumination strategy spend a long time thinking about and analyzing their condition. Such a situation could develop a kind of immobility in students that potentially leads to avoiding from engaging in active problem-solving and to avoidance strategies in general (Moulds et al., 2007).

In addition to ED, our results suggested that SC predicts SA significantly, which is in line with previous studies (Wentzel, 1991; Elias and Haynes, 2008). Studies have repeatedly suggested that SC is linked to social adjustment abilities and the ability to make use of environmental and personal resources to achieve a desired social outcome (Hussong et al., 2005; Brown et al., 2008; McElhaney et al., 2008; Lee et al., 2010). Poor SC is associated with the lack of proper social skills (e.g., being inattentive and unprepared during instructional periods, aggressive behavior toward classmates and educational staff, inability to engage in cooperative learning, and disruptive behavior in the classroom), which negatively affects students’ academic skills (studying skills, problem-solving skills, critical and decision-making skills, mastery and performance skills, task management skills). Additionally, students with less social skills face more disciplinary consequences when they fail to engage in appropriate behavior (Martens and Witt, 2004; Eleby, 2009). In conclusion, poor SC leads to chaotic relationships in school with students and teachers, which is among the etiological factors of SA (Legault et al., 2006).

Finally, the current study replicated the influence of peer relationships on SA, which has been proposed in previous studies (e.g., Schmidt et al., 2019). The degree to which children feel socially integrated and accepted in the classroom defines their school achievement *via* the accessibility of social support or inclusion in working groups (Ladd et al., 2012). Also, feeling related to peers is associated with high positive emotions at school and home on a daily basis, representing the importance of peer relatedness in promoting positive well-being (Schmidt et al., 2019). Youth who feel a strong attachment to their schools and peers show improved academic outcomes (Nasir et al., 2011), enhanced self-efficacy (Murphy and McKenzie, 2016), reduced depressive symptoms (Joyce and Early, 2014), higher commitment to school (Libbey, 2004), and a greater sense of safety in the school environment (Ethier et al., 2018), which decrease the likelihood of the development of SA. On the other hand, those with low attachment to peers and schools are more likely to withdraw from their education (Finn, 1989).

In sum, the current study results indicated that ED, SC, and PR significantly predict SA. These findings suggest that school psychologists and other clinicians design interventions to improve the students’ SD, ER, and PR shortcomings.

The current study results should be interpreted with respect to a few limitations. First, our study sample included only girls. There are significant differences between males and females, especially in emotional abilities (e.g., McRae et al., 2008; Deng et al., 2016). Thus, the results of the current study could not be generalized to male samples. Second, the small sample size of the current study and the fact that we only included students from schools located in one city limits the generalization of the results. Third, for the data gathering, we used only self-report measures, so it is possible that correlations between self-report measures may partly be explained by shared method variance. Finally, the cross-sectional nature of the current study does not allow conclusions about causality and prognosis (e.g., ED, SC, and PR as a predictor of future SA).

## Data Availability Statement

The raw data supporting the conclusions of this article will be made available by the authors, without undue reservation.

## Ethics Statement

The studies involving human participants were reviewed and approved by the ethics committee of the Islamic Azad University, Sarab Branch. Written informed consent to participate in this study was provided by the participants’ legal guardian/next of kin.

## Author Contributions

ZV and MA gathered data and prepared the manuscript. MEA performed the data analyses and reviewed and revised the manuscript. All authors contributed to the article and approved the submitted version.

## Conflict of Interest

The authors declare that the research was conducted in the absence of any commercial or financial relationships that could be construed as a potential conflict of interest.

## Publisher’s Note

All claims expressed in this article are solely those of the authors and do not necessarily represent those of their affiliated organizations, or those of the publisher, the editors and the reviewers. Any product that may be evaluated in this article, or claim that may be made by its manufacturer, is not guaranteed or endorsed by the publisher.
